# Arterial Monitoring in Hypertensive Emergencies: Significance for the Critical Care Resuscitation Unit

**DOI:** 10.5811/westjem.59373

**Published:** 2023-07-17

**Authors:** Quincy K. Tran, Dominique Gelmann, Manahel Zahid, Jamie Palmer, Grace Hollis, Emily Engelbrecht-Wiggans, Zain Alam, Ann Elizabeth Matta, Emily Hart, Daniel J. Haase

**Affiliations:** * University of Maryland School of Medicine, Baltimore, Maryland; † University of Maryland School of Medicine, Program in Trauma, The R Adams Cowley Shock Trauma Center, Baltimore, Maryland; ‡ Department of Emergency Medicine, University of Maryland School of Medicine, Baltimore, Maryland; § University of Maryland School of Medicine, Department of Emergency Medicine, The Research Associate Program in Emergency Medicine and Critical Care, Baltimore, Maryland

## Abstract

**Introduction:**

Blood pressure measurement is important for treating patients. It is known that there is a discrepancy between cuff blood pressure vs arterial blood pressure measurement. However few studies have explored the clinical significance of discrepancies between cuff (CPB) vs arterial blood pressure (ABP). Our study investigated whether differences in CBP and ABP led to change in management for patients with hypertensive emergencies and factors associated with this change.

**Methods:**

This prospective observational study included adult patients admitted between January 2019–May 2021 to a resuscitation unit with hypertensive emergencies. We defined clinical significance of discrepancies as a discrepancy between CBP and ABP that resulted in change of clinical management. We used stepwise multivariable logistic regression to measure associations between clinical factors and outcomes.

**Results:**

Of 212 patients we analyzed, 88 (42%) had change in management. Mean difference between CBP and ABP was 17 milligrams of mercury (SD 14). Increasing the existing rate of antihypertensive infusion occurred in 38 (44%) patients. Higher body mass index (odds ratio [OR] 1.04, 95% confidence Interval [CI] 1.0001–1.08, *P*-value <0.05) and history of peripheral arterial disease (OR 0.16, 95% CI 0.03–0.97, *P*-value <0.05) were factors associated with clinical significance of discrepancies.

**Conclusion:**

Approximately 40% of hypertensive emergencies had a clinical significance of discrepancy warranting management change when arterial blood pressure was initiated. Further studies are necessary to confirm our observations and to investigate the benefit-risk ratio of ABP monitoring.

## INTRODUCTION

Blood pressure (BP) monitoring is an essential component of managing hypertensive disease processes, many of which require maintenance of specific BP windows. Intra-arterial blood pressure (ABP) monitoring is widely considered the gold standard of BP measurement in critical care settings; however, its invasive nature also presents some rare but serious risks including bleeding, thrombosis, infection, embolism, and nerve damage.^1^,^2^ Additionally, little research has been done to demonstrate whether its use is associated with changes in clinical management when compared to noninvasive cuff blood pressure (CBP) monitoring, such as oscillometric CBP measurement. Therefore, the necessity of ABP monitoring has been questioned.^3^,^4^

Previous studies have shown, however, that discrepancies exist between noninvasive CBP and invasive ABP measurement.^5^–^12^ Accuracy of CBP measurement may be affected by obesity, cuff location, age, and arterial stiffness.^13^–^16^ Accurate measurement of BP is important, as brief episodes of either hypotension or hypertension are associated with higher rates of mortality and other unfavorable outcomes in various critical illnesses.^17^–^22^ Use of CBP generally underestimates intra-arterial systolic (SBP) in hypertensive diseases, which may lead to mismanagement of patients with conditions requiring adherence to specific BP guidelines.^6^,^10^,^23^–^25^ Additionally, ABP monitoring offers the benefit of real-time continuous monitoring, while cuff measurements are typically performed intermittently.

Guidelines established by several professional societies recommend specific BP goals for various hypertensive disease states. For example, current guidelines for management of acute aortic diseases suggest a SBP goal of <120 millimeters of mercury (mm Hg) in Type A dissection and SBP goal of <140 mm Hg in Type B dissection.^26^ Additional guidelines exist for ischemic stroke,^27^ spontaneous intracerebral hemorrhage,^28^ and preeclampsia,^29^ among others. As previously discussed, operating within a safe BP margin has important clinical implications. For example, in patients with acute aortic pathology, both hypotension and hypertension are associated with increased mortality risk.^18^,^21^,^22^ This highlights the importance of accurate continuous BP measurement to maintain pressures within specific parameters.

A few previous studies provide evidence for the utility of ABP monitoring in hypertensive critically ill patients. Manios et al found that CBP measurements underestimate ABP in patients with ischemic stroke, most markedly in those with critically high SBP.^11^ A retrospective study by Raffman et al demonstrated that nearly one in three patients with hypertensive disease admitted to a resuscitation unit had a difference between ABP and CBP that would result in a change in clinical management.^6^ However, these studies were limited by small sample size or the use of hypothetical definitions of change in management, respectively.

Our prospective study sought to investigate the clinical significance of the difference between ABP monitoring vs CBP monitoring, in real time, among patients with hypertensive critical illnesses. We specifically aimed to determine whether the difference in monitoring between CBP and ABP would result in change in clinical management for patients with hypertensive emergencies and to identify clinical factors predicting change in management between the two measurement modalities. We hypothesized that the difference between monitoring of ABP and CBP values would lead to a change in BP management in at least 30% of our patients with hypertensive emergencies.

**Population Health Research Capsule**

What do we already know about this issue?

*Discrepancy between arterial (ABP) vs cuff blood pressure (CBP) monitoring exists. CBP is commonly used although ABP monitoring is considered the gold standard*.

What was the research question?

*Does a discrepancy between the two blood pressure monitoring modalities result in change of clinical management?*

What was the major finding of the study?

*88 patients (42%) had a change of management in real time resulting in increasing doses of antihypertensive medication*.

How does this improve population health?

*In a patient with hypertensive emergency whose blood pressure is at borderline of recommended guidelines, ABP should be considered*.

## METHODS

### Study Setting

This prospective observational study took place in the critical care resuscitation unit (CCRU), an intensive care unit (ICU)-based resuscitation unit that was created at our quaternary-care institution with the goal of expediting transfer of patients with time-sensitive critical illnesses from other hospitals when there are no available beds at one of our traditional ICUs.^30^,^31^ These patients’ critical illnesses usually exceed the capability of the sending hospitals; so, upon their arrival at the CRRU, they receive immediate resuscitation and timely ICU-level care. Once patients are stabilized, they are moved to appropriate inpatient beds. Per CCRU clinical policy, all patients receiving antihypertensive medication infusion or requiring hemodynamic monitoring will need ABP monitoring, and an arterial catheter is placed upon arrival to the unit. The CCRU clinicians would place a majority of arterial cannulations, in compliance with our institutional protocols for maximum sterility. The study was approved by our institutional review board (HP-00079864) and was exempted from formal consent.

### Patient Selection

We included consecutive adult patients with any hypertensive emergency diagnosis (ischemic stroke, spontaneous intracerebral hemorrhage, acute aortic diseases) who required ABP monitoring upon admission to the CCRU between January 2019–May 2021. Patients who had CBP and ABP measurements within 60 minutes of each other were eligible. Exclusion criteria included the following: arterial catheter in place prior to arrival; arterial catheter and BP cuff placement on opposite side of body (ie, cuff pressure on left arm while arterial catheter in right radial artery); unreliable arterial measurements according to the clinicians; use of vasopressors prior to or starting at the time of arterial catheter placement. We additionally excluded patients with non-hypertensive diagnoses (eg, sepsis, organ ischemia, pancreatitis, any bleeding, respiratory failure). We excluded these patients because their BPs are managed differently. Patients who require vasopressors or have nonhypertensive diagnoses are managed in accordance with their mean arterial pressure, instead of SBP, as recommended by previous management guidelines for hypertensive emergencies.

### Prospective Data Collection

Prior to the study recruitment period, we created a standardized form and educated the CCRU clinicians (nursing staff, advanced practice practitioners, residents, fellow physicians) on the use of the form for all patients requiring arterial catheter placement. Most of the CCRU nursing staff were blinded to the study hypothesis and were not involved in preparation of the manuscript. The form contained sections for clinicians to record patient demographic information (age, gender, medical record number, diagnosis, goal of BP), CBP values, ABP values, arterial catheter site (right/left radial or femoral), cuff site (right/left arm or leg), and management decisions as guided by each BP value.

Before placing the arterial catheter, the CCRU nurse or the clinician prospectively filled out the form in real time to indicate CBP values immediately and the associated management according to the CBP monitoring values (eg, decrease nicardipine infusion from 7.5 milligrams per hour [mg/hr] to 5 mg/hr). After arterial catheter placement, the clinicians additionally recorded the ABP monitoring values and the management decision guided b those values (eg, increase nicardipine infusion from 7.5 mg/hour to 10 mg/hour, etc). An APP and the principal investigator adjudicated the missing data on the prospective data collection form, using patients’ electronic health records (EHR). We identified approximately 23 patients (11%) who did not have a diagnosis that required ABP monitoring in the CCRU. These included 14 patients with aortic aneurysms and nine other diagnoses.

### Retrospective Data Collection

Members of the research team retrospectively collected additional patient data from our institution’s EHR. Extracted demographic information included age, gender, body mass index (BMI), and past medical history. They also collected clinical data during hospitalization, including lab values (serum creatinine and lactate), echocardiography results (ejection fraction, presence of left ventricular hypertrophy), medications used at the time of arterial cannulation, and patient outcomes (deceased, discharged to home, discharged to acute rehabilitation facility, etc). Additionally, they collected information regarding complications associated with arterial catheter cannulations and total duration of arterial catheter insertion. We defined arterial catheter complications as bleeding, aneurysm, extremity necrosis, local nerve damage, or definitive source of local or systemic infection or embolism.^32^ We had planned to impute any missing retrospective data with the population’s mean; however, no data was missing as all the recorded data was part of the clinical standard of care.

We obtained all retrospective data in compliance with methodologic standards for medical record review.^33^ Members of the research team were not blinded to the study hypothesis. Each investigator received training in data extraction from patient records and input data into a standardized Excel spreadsheet (Microsoft Corporation, Redmond, WA). To reduce bias, investigators collected specific subsets of data only; for example, those who recorded hospital outcomes did not collect BP measurements or lab values and vice versa. The senior investigator reviewed the accuracy of data during the training phase to ensure greater than 90% agreement before team members started data collection. Additionally, the senior investigator randomly rechecked up to 20% of the collected data to ensure accuracy throughout the process.

### Outcome Measures

Our primary outcome was the prevalence of clinically significant difference between measurements of ABP and CBP monitoring. We defined this as a difference in clinical management based on the values obtained from ABP vs CBP monitoring, as determined in real time by the clinicians upon placement of the arterial catheters. For example, the American Heart Association Task Force on Practice Guidelines recommends a SBP of ≤120 mm Hg for type A aortic dissection.^26^ We identified clinically significant difference as a difference between ABP and CBP that resulted in a change in BP management to adhere to these guidelines.

If a patient with Type A aortic dissection had CBP of 115 mm Hg and ABP of 125 mm Hg, the difference in BP measurements necessitated a change in management between the two modalities. In this case, the CBP value indicated the SBP was at goal and no further action was needed. In contrast, the ABP suggested that increasing the dose of the current antihypertensive infusion was warranted. Alternatively, if the patient had CBP of 135 mm Hg and ABP of 145 mm Hg, the clinician indicated that both values required increasing the dose of current antihypertensive infusion, in accordance with the guidelines to lower SBP to ≤120 mm Hg. Thus, this action indicated that no clinically significant difference existed.

Our secondary outcomes included mean difference between CBP monitoring and ABP monitoring and the percentage of patients with difference of ≥20 mm Hg between the two modalities. Given that previous guidelines suggested that patients with a difference of ≥20 mm Hg may be at risk for worse outcomes than those with a difference ≤20 mm Hg,^34^ we determined this cutoff to be a significant difference in this study. We additionally reported factors associated with either the primary or secondary outcomes.

### Sample Size Calculation

We calculated our sample size according to a prior study from Ruszala.^10^ Based on this study, which reported that ABP monitoring was associated with approximately 20% higher prevalence of patients who received more frequent interventions during transport, we calculated that we would need 97 patients in each group (total 194 patients) to detect a difference of 20% of prevalence of clinical management between CBP and ABP monitoring, with a = 0.05 and power of 80%.

### Data Analysis

We used descriptive analysis to present patient data. Categorical variables are presented as percentages, and continuous variables are reported as mean (+/− SD) or median (interquartile range), as applicable. We analyzed differences between groups of continuous variables using the Student *t*-test or Mann Whitney U test, while the chi-square test was used for categorical variables.

To graphically represent the distributions of ABP and CBP monitoring differences, we used the Bland-Altman analysis. The Y-axis of the Bland-Altman graphs depicted the values of [ABP-CBP] differences. If ABP values are >CBP values, there would be more dots in the positive region of the Y-axis. The X-axis represented the ranges of patients’ CBP monitoring values. A dispersed distribution along the X-axis would suggest that the differences between [ABP-CBP] occurred at all ranges of CBP monitoring values.

We used forward stepwise multivariable binary logistic regressions to identify associations between independent variables and the outcomes (clinically significant difference, ABP-CBP difference >20 mm Hg). We a priori determined the independent variables included in regression analysis, which are listed in Appendix 1. Goodness-of-fit, multicollinearity, and discriminatory capability of the multivariable logistic regression models were also assessed. A Hosmer-Lemeshow test with *P*-value > 0.05 indicates a model with a good fit of independent variables. We reported the variance inflation factors (VIF) for assessment of the multicollinearity of independent variables, and factors with VIF ≥5 were considered to have high multicollinearity and thus were removed from the logistic regression. The discriminatory capability of the regressions was assessed via the area under the receiver operating curve (AUROC). Models with AUROC approaching −1 or +1 were considered having perfect discriminatory capability between dichotomous outcomes.

We performed all statistical tests using Minitab version 19.0 (Minitab Corp, State College, PA). A *P*-value of less than 0.05 was considered statistically significant.

## RESULTS

### Patient Characteristics

**Figure 1:**
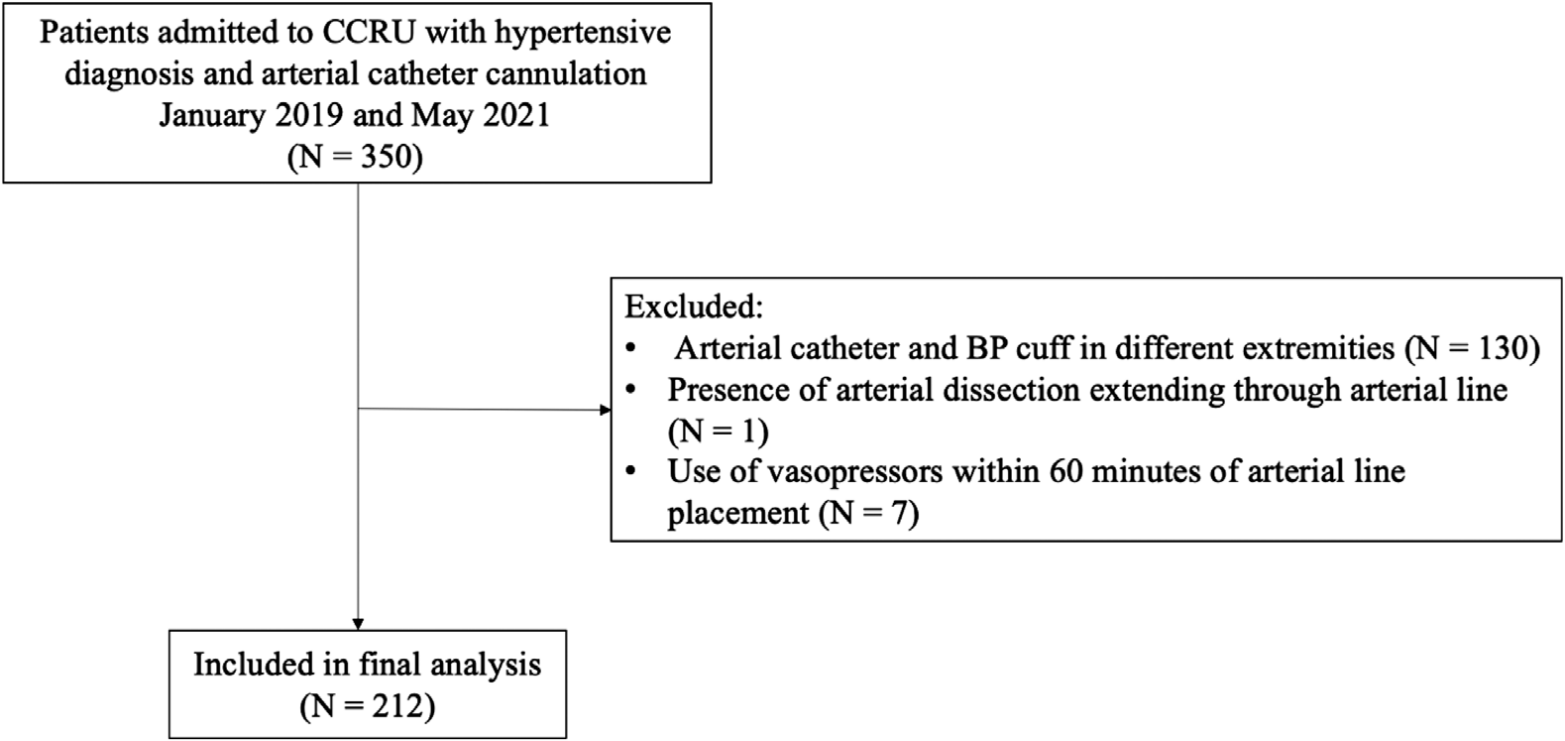
Patient selection diagram. A total of 212 patients with hypertensive disease and arterial catheter cannulation were included in analysis. *CCRU*, critical care resuscitation unit; *BP*, blood pressure.

We identified a total of 350 patients with hypertensive diagnoses and arterial catheter cannulation for ABP monitoring between January 2019–May 2021 (Figure 1). Among these patients, 212 met our inclusion criteria and were included in analysis. A total of 88 patients (42%) had a change of management in real time (Table 1). There was only one (0.5%) complication from arterial catheter use among the entire group of patients (Table 2). Other hospital outcomes were similar between the groups.

Of the included patients, the most frequent hypertensive diagnoses included spontaneous intraparenchymal hemorrhage (22%), type A dissection (20%), type B dissection (16%), and subarachnoid hemorrhage (14%) (Table 1). Baseline clinical characteristics (age, BMI, serum lactate, left ventricular ejection fraction, presence of ventricular hypertrophy) were similar between patients with change in management and those without. Additionally, there were no statistically significant differences between the two groups with respect to pre-existing medical conditions, arterial catheter location, or time in minutes between ABP and CBP measurement.

### Difference Between Arterial Blood Pressure-Cuff Blood Pressure

The mean difference between ABP and CBP in the overall study population was 17 mm Hg (SD 14), with a mean difference of 11 mm Hg (SD 10) in the group without clinically significant BP difference and 26 mm Hg (SD 14) in the group with clinically significant difference (Table 2). Seventy-eight patients (37%) had a difference in BP of ≥20 mm Hg between the two modalities.

**Figure 2:**
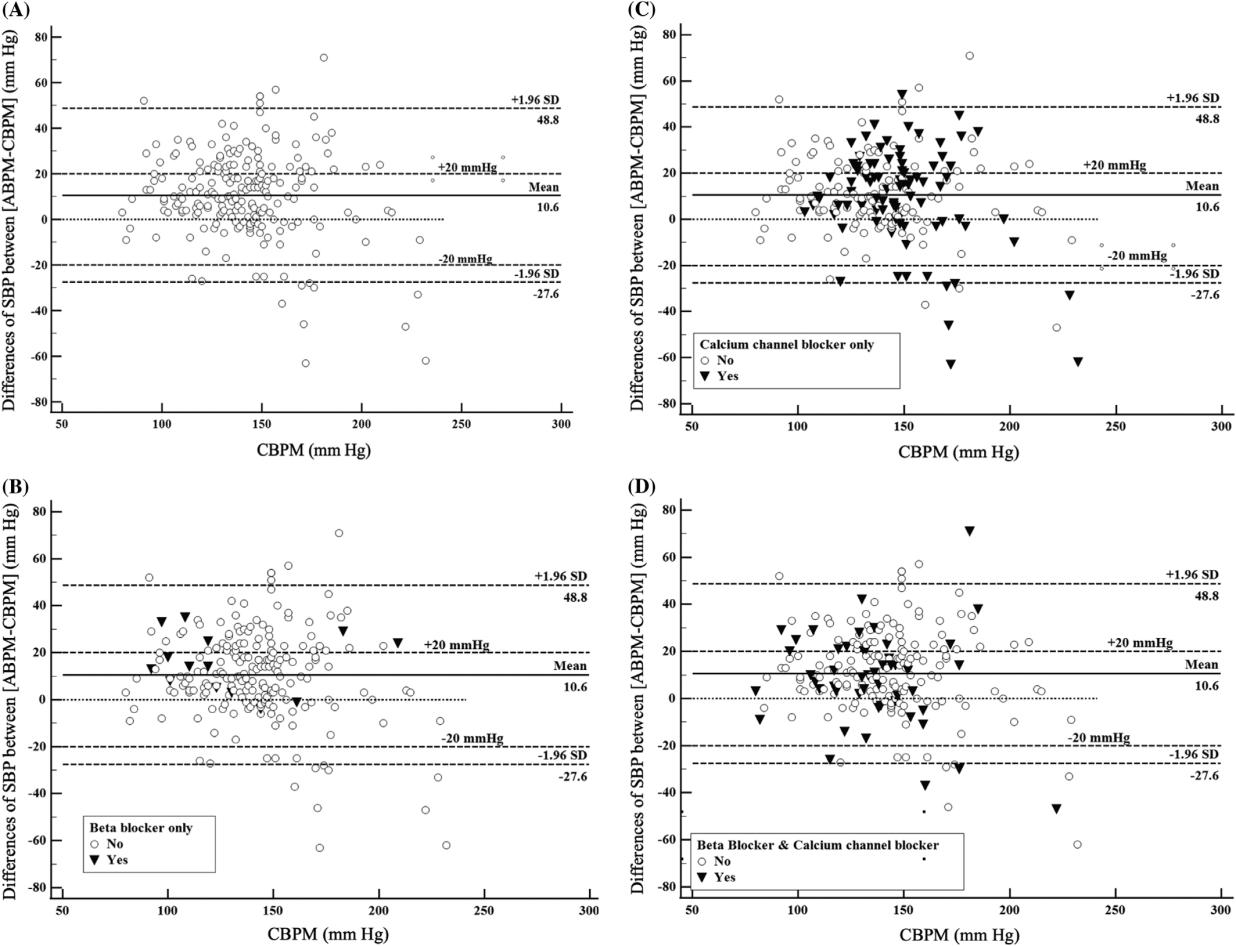
(A) Bland-Altman graph depicting the difference between initial arterial blood pressure and cuff blood pressure monitoring values for all hypertensive patients. (B–D) Bland-Altman graph comparing initial values of arterial blood pressure and cuff blood pressure monitoring values for hypertensive patients who received or did not receive (B) beta blocker antihypertensive medication alone for antihypertensive therapy; (C) calcium channel blocker antihypertensive medication alone for antihypertensive therapy; and (D) both beta blocker and calcium channel blocker antihypertensive medication. ABPM, arterial blood pressure monitoring; CBPM, cuff blood pressure monitoring; mm Hg, millimeters of mercury. *ABPM*, arterial blood pressure monitoring; *CBPM*, cuff blood pressure monitoring; *mm Hg*, millimeters of mercury.

The Bland-Altman graph illustrating [ABP-CBP] differences among the included patients demonstrated that the mean difference between the two BP measurements was 10.6 mm Hg (Figure 2A). The graph also showed that a large percentage of patients had ABP monitoring values >CBP monitoring values. These trends were observed among patients who received beta-blocker infusions (Figure 2B), calcium channel blocker infusions (Figure 2C), or both beta-blocker and calcium channel blocker infusions (Figure 2D). Similarly, among these patients with hypertensive emergencies, a large percentage had [ABP-CBP] differences ≥20 mm Hg, compared to the number of patients who had [ABP-CBP] differences ≥−20 mm Hg.

### Clinically Significant Difference

Within our study population, 88 of 212 patients (42%) had a clinically significant difference between CBP and ABP monitoring as demonstrated by a change in management between the two measurement modalities (Table 2). Of patients with a change in management as guided by ABP vs CBP monitoring, 44% required an increase in antihypertensive medication dose per ABP but not per CBP values, and 40% warranted a new antihypertensive medication according to ABP but not CBP monitoring. Another 6% required a decreased dose of antihypertensive infusion per ABP but not per CBP monitoring, and 9% required maintenance of an antihypertensive infusion per ABP monitoring when CBP monitoring indicated discontinuation of the infusion (Table 3).

Of patients who did not have a change in management between ABP and CBP monitoring, 64% required continuation of the current regimen, 23% required an increased dose of current antihypertensive infusion or an addition of a new medication per both values, and 2% warranted a decreased dose per both CBP and ABP monitoring (Table 3). In 11% of patients, both CBP- and ABP-monitored measurements warranted initiation of a new antihypertensive medication.

### Predictors of Change in Management

Our multivariable regressions identified that each unit increase of BMI was associated with 4% higher likelihood of having a change in management among patients with hypertensive emergencies (odds ratio [OR 1.04, 95% confidence interval [CI] 1.00–1.08, *P*-value <0.05). Patients who had history of peripheral arterial disease (OR 0.16, 95% CI 0.03–0.97, *P*-value <0.05)were also associated with having lower likelihood of a clinically significant difference between ABP and CBP monitoring (Table 4). This model had an acceptable discriminatory capability with AUROC of 0.64. Additionally, a multivariable logistic regression identified that right-sided arterial catheters were associated with significantly lower odds for [ABP-CBP monitoring] difference ≥20 mm Hg (OR 0.46, 95% CI 0.25–0.85, *P*-value <0.05) (Table 4). The model also had acceptable AUROC of 0.67.

## DISCUSSION

While two retrospective studies have demonstrated clinically relevant differences in CBP and ABP measurements,^6^,^12^ to our knowledge this is the first prospective study investigating the clinical relevance of differences between CBP and ABP monitoring among patients with different diagnoses of hypertensive emergencies. The results of this observational study support our hypothesis that the discrepancy between ABP and CBP measurements leads to a change in BP management in a large percentage of patients with hypertensive emergencies.

In this study, CBP monitoring underestimated ABP monitoring (by 17 mm Hg on average), and measurement of the latter warranted increases in antihypertensive medication. This suggests that the use of CBP monitoring alone to guide management of patients with hypertensive emergencies may result in unrecognized and untreated levels of hypertension. Barton et al suggested that patients with spontaneous intracerebral hemorrhage who sustained hypertension during the acute phase in the emergency department (ED) were associated with poor neurological outcome at one-month and 12-month follow-ups.^35^ Similarly, patients with acute uncomplicated type B aortic dissection but who continue to have hypertension were associated with higher in-hospital mortality, compared to those who have controlled BP.^36^

Although patient outcomes (which were not the outcomes in this observational and exploratory study) in univariate analyses were not different between groups with or without clinically relevant differences, clinicians should thus consider the use of ABP monitoring in hypertensive patients for whom adherence to specific BP goals is important. Additionally, when a patient is having BP at the borderline recommended by current guidelines, coupled with prolonged boarding time in the ED, the addition of ABP monitoring would provide helpful information for further medical decision-making, as it has been shown that CBP monitoring is not as reliable.^37^ Future studies should further investigate our observation to better characterize the association between CBP and ABP monitoring differences and patient outcomes.

The use of invasive ABP monitoring is not entirely without risk and expense. Risks of ABP monitoring include bleeding, thrombosis, infection, embolism, and nerve damage; however, the incidence of complications is low with major complications occurring in less than 1% of cases.^3^,^4^,^33^ We found one reported complication of soft hematoma within our study sample of 212 patients, producing an overall complication rate of 0.5%. On the other hand, we found a change in management rate of 42% in patients with the use of ABP monitoring. Thus, the potential benefit of ABP monitoring in guiding clinical management appeared to outweigh the risks of harm in our patients.

The use of ABP monitoring does incur additional cost of care. At our institution, the one-time, per-patient supply cost for initiation of ABP monitoring is approximately $55 US dollars (USD). With a change in management rate of 42% among our patients upon insertion of an arterial catheter, ABP monitoring was able to detect one change in management for approximately every three patients. The total cost to detect one clinically significant BP difference for every three patients with hypertensive critical illness is, therefore, $165 USD. This observational and exploratory study was not designed to detect differences in patient outcomes such as mortality, ICU or hospital length of stay, and did not demonstrate a direct benefit for ABP monitoring in the study patient population. Further studies should investigate the benefits-risk ratio between potential benefits vs financial cost and complications between ABP and CBP monitoring.

Within our patient population, patients with clinically significant difference between CBP and ABP monitoring had higher BMI than those without a clinically significant difference (30.1 vs 28.2, respectively), although the difference was not statistically significant. Previous studies have identified an association between obesity and greater differences between ABP and CBP monitoring, with CBP measurements typically underestimating direct intra-arterial SBP.^13^,^14^ This is thought to be due to the arm circumference of obese patients preventing proper fit of BP cuffs, although a definitive cause has not been identified.^13^,^14^

Our finding that patients with peripheral artery disease were associated with lower odds of change in clinical management appeared counterintuitive at first. However, a study by Iida et al suggested that patients with arterial stiffness would have higher BP than those who did not.^16^ Therefore, it would be probable that *both* ABP and CBP monitoring values from patients with peripheral artery disease, and their resulting arterial stiffness, would be higher than the recommended goal of SBP by guidelines. Thus, both ABP and CBP monitoring values would recommend an increase of current antihypertensive infusion, which is not considered a change in clinical management by our definition. However, our patient population included only six (3%) patients with peripheral artery disease; further studies with higher percentages of patients with peripheral artery disease are necessary to confirm our observations. The result from our multivariable logistic regression also showed that ABP monitoring on the right side was associated with lower likelihood of greater differences between CBP and ABP. While our study could not explain these findings, it could be a confounding finding or it could be due to the number of patients with acute aortic dissection within our patient population. Patients with acute aortic dissection have been shown to have greater differences between both arms, with BP on the left arm being affected more.^38^ This phenomenon would have caused bigger differences between CBP and ABP on the left side. However, further studies including only patients with acute aortic diseases are needed to confirm or refute our observation.

Although identification of specific subsets of patients who would most benefit from ABP monitoring may be helpful in directing the placement of arterial catheters in the acute stages of illness, our study revealed very few predictors of such. The high incidence of clinically relevant ABP-CBP discrepancy and lack of clear distinction as to which patients are more likely to require different BP management after placement of an arterial catheter suggest that clinicians should consider invasive ABP monitoring more often in patients with hypertensive emergencies requiring a continuous antihypertensive infusions, at least during the acute resuscitation phase.

## LIMITATIONS

Our study has several limitations, the first having to do with the clinical setting and the patient population. Although most of these disease states are encountered in the ED setting, the results in our setting may not be applicable to many EDs. Thus, our study may lack external validity. Additionally, we did not address any downstream consequences, such as clinical outcomes and throughputs that could have been derived from the change of clinical management of these patients, as this topic was beyond the scope of this study. The heterogeneity of our patient population with respect to hypertensive emergency diagnosis limits our ability to draw conclusions about specific subsets of hypertensive disease. Because intra-arterial BP monitoring is considered the gold standard, we did not randomize patients to management guided by ABP vs CBP monitoring to determine associations between each measurement modality and patient outcomes. Finally, our limited sample size may have prevented accurate depiction of the incidence of arterial catheter complications or patient-related outcomes. Despite these limitations, our study does provide further evidence to support the use of invasive arterial catheters for BP monitoring in patients with hypertensive emergencies and requiring continuous antihypertensive infusions.

## CONCLUSION

Approximately 4 in 10 patients with hypertensive emergencies had a change in management between monitoring of cuff blood pressure vs arterial blood pressure monitoring, indicating clinical relevance of the discrepancy in BP values obtained by these two measurement modalities. Patients with high BMI were associated with higher likelihood of requiring change of management due to the discrepancies between ABP and CBP monitoring. Additional studies and less heterogenous patient pathologies are recommended to further explore patient outcomes associated with these findings. Further studies are necessary to confirm our observations and to investigate the benefit-risk ratio of arterial blood pressure monitoring.

## Supplementary Information



## Figures and Tables

**Table 1:** Characteristics of hypertensive patients with change in management vs patients without change in management between intraarterial and noninvasive blood pressure monitoring.

Demographic	All patients N = 212	No change in management N = 124 (58.5%)	Change in management N = 88 (41.5%)	*P*-value
Age, mean (SD)	63 (14)	62 (13)	62 (15)	0.9
Gender, N (%)				
Male	130 (61)	77 (62)	53 (60)	0.8
Female	82 (39)	47 (38)	35 (40)	0.8
BMI, mean (SD)	29.0 (6.7)	28.2 (6.0)	30.1 (7.4)	0.4
Clinical data				
Serum lactate, mean (SD)	2.1 (1.6)	2.1 (1.7)	2.1 (1.3)	0.8
Left ventricular EF, mean (SD)^1^	0.60 (0.12)	0.60 (0.12)	0.60 (0.12)	0.9
Left ventricular hypertrophy, N (%)	128 (60)	74 (60)	54 (62)	0.7
Arterial catheter location, N (%)				
Right	126 (59)	75 (60)	51 (58)	0.7
Left	86 (41)	49 (40)	37 (42)	0.7
Radial	209 (99)	121 (98)	88 (100)	N/A^2^
Other (brachial, femoral)	3 (1)	3 (2)	0 (0)	N/A^2^
Time between IABP and NIBP measurement in minutes, mean (SD)	1.2 (2.3)	1.0 (2.0)	1.4 (2.7)	0.7
Pre-existing conditions, N (%)				
Diabetes mellitus	51 (24)	28 (23)	23 (26)	0.6
Hypertension	177 (84)	101 (82)	76 (86)	0.3
Coronary artery disease	25 (12)	16 (13)	9 (10)	0.6
Peripheral arterial disease	6 (3)	2 (2)	4 (5)	0.2
Kidney disease	37 (18)	23 (19)	14 (16)	0.6
Obesity	83 (39)	44 (36)	39 (44)	0.2
Diagnosis, N (%)				
Aortic aneurysm	14 (7)	11 (9)	3 (3)	0.09
Stroke without TPA	19 (9)	10 (8)	9 (10)	0.6
Stroke with TPA	18 (9)	10 (8)	8 (9)	0.8
Subarachnoid hemorrhage	30 (14)	18 (15)	12 (14)	0.9
Intracerebral hemorrhage with IVH	28 (13)	14 (11)	14 (16)	0.3
Intracerebral hemorrhage without IVH	19 (9)	8 (7)	11 (13)	0.1
Type A aortic dissection	42 (20)	27 (22)	15 (17)	0.4
Type B aortic dissection	33 (16)	21 (17)	12 (14)	0.5
Other^3^	9 (4)	5 (4)	4 (5)	0.9

1 Transthoracic echocardiogram was available for all patients in the analysis.

2 We did not perform a statistical analysis due to the presence of zero counts in this subgroup.

3 Other diagnoses included patients with three iliac aneurysms, two renal artery aneurysms, two nonspecific hypertensive emergencies, pre-eclampsia, and one non-ST-elevation myocardial infarction.

*BMI*, body mass index; *EF*, ejection fraction; *IABP*, intra-arterial blood pressure; *IVH*, intraventricular hemorrhage; *LVEF*, left ventricular ejection fraction; *LVH*, left ventricular hypertrophy; *NIBP*, noninvasive blood pressure; *TPA*, tissue plasminogen activator.

**Table 2:** Blood pressure values, interventions, and clinical outcomes among patients admitted to the critical care resuscitation unit with hypertensive disease. Higher arterial blood pressure (ABP) values were associated with change in management between ABP and cuff blood pressure monitoring.

	All patients N = 212	No change in management N = 124 (58.5%)	Change in management N = 88 (41.5%)	*P*-value
Blood pressure values				
ABP SBP (mm Hg), *mean (SD)*	145 (23)	145 (27)	162 (34)	<**0.001**
CBP SBP (mm Hg), *mean (SD)*	138 (24)	139 (28)	145 (28)	0.2
ABP-CBP dfference (mm Hg), *mean (SD)*	17 (14)	11 (10)	26 (14)	<**0.001**
Patients with ABP-CBP difference ≥20 mm Hg, *N (%)*	78 (37)	24 (19)	54 (61)	<**0.001**
Clinical interventions				
Mechanical ventilation, *N (%)*	57 (27)	32 (26)	25 (28)	0.7
Medication, *N (%)*				
Beta blocker	62 (29)	37 (29)	25 (28)	0.8
Calcium channel blocker	134 (63)	75 (60)	59 (67)	0.3
Both beta and calcium channel blocker	49 (23)	29 (23)	20 (23)	0.9
>1 Beta blocker	2 (1)	1 (1)	1 (1)	0.8
>1 Calcium channel blocker	8 (4)	5 (4)	3 (3)	0.8
Propofol	38 (18)	25 (20)	13 (15)	0.3
Fentanyl	60 (28)	38 (31)	22 (25)	0.4
Patients with arterial line complication, N (%)	1 (0.5)	0 (1.1)	1 (0)	N/A^1^
Hospital course (days), *median [IQR]*				
Length of arterial catheter placement	2 [1–4]	2 [1–3]	2 [1–4]	0.1
Hospital length of stay	10 [5–18.25]	10 [5–19.25]	9.5 [5–17]	0.96
ICU length of stay	6 [3–12.25]	6 [3–13]	6 [3–11]	0.9
Discharge destination, *N (%)*				
Home, self-care	38 (18)	20 (16)	18 (20)	0.4
Home health care, acute care, or rehabilitation center	133 (63)	82 (66)	51 (58)	0.2
Skilled nursing facility	11 (5)	6 (5)	5 (6)	0.98
Deceased/Hospice	31 (15)	16 (13)	15 (17)	0.4

1 We did not perform a statistical analysis due to the presence of zero counts in this subgroup.

*ABP*, arterial blood pressure; *ABPM*, arterial blood pressure monitoring; *CBP*, cuff blood pressure; *CBPM*, cuff blood pressure monitoring; *ICU*, intensive care unit; *mm Hg*, millimeters of mercury; *SBP*, systolic blood pressure; IQR, interquarttile range.

**Table 3:** Management decisions for hypertensive critically ill patients with and without clinically significant difference between arterial blood pressure and cuff blood pressure measurement.

Type of BP management intervention	Number of patients (%)
Patients with change in clinical management according to ABP vs CBP measurement, N = 88	
Increase existing medication dose	38 (44)
Decrease existing medication dose	7 (6)
Add new antihypertensive medication	35 (40)
Maintain infusion versus discontinuing	8 (9)
Patients without change in clinical management between ABP vs CBP measurement, N = 124	
Continue existing regimen (no changes)	79 (64)
Both ABP and CBP warranted an increased antihypertensive dose	29 (23)
Both ABP and CBP warranted a decreased antihypertensive dose	3 (2)
Both ABP and CBP warranted initiation of new antihypertensive medication	13 (11)

*ABP*, arterial blood pressure; *BP*, blood pressure; *CBP*, cuff blood pressure.

**Table 4:** Stepwise multivariable logistic regression for identification of clinical factors associated with clinically significant difference between arterial blood pressure and cuff blood pressure measurements. Predetermined factors were entered into models and only factors with a significant association were reported. Models for each outcome measure showed both food fit of independent variables and good discriminatory capability (higher AUROC).

	OR	95% CI	*P*-value
Primary outcome: clinically significant difference between ABP and CBP^1^			
Body mass index	1.04	(1.001, 1.084)	0.045
Peripheral arterial Disease	0.16	(0.03–0.97)	0.046
Secondary outcome: difference between ABP and CBP ≥20 mm Hg^2^			
Right-sided arterial catheter location	0.46	(0.25, 0.85)	0.013

1 Hosmer-Lemeshow DF = 8; chi-square = 14.12, *P*-value = 0.079. AUROC = 0.64.

2 Hosmer-Lemeshow DF = 8; chi-square = 7.04, *P*-value = 0.532. AUROC = 0.67.

*ABP*, arterial blood pressure; *AUROC*, area under the receiver operating characteristic curve; *CBP*, cuff blood pressure; *CI*, Confidence Interval; *mmHg*, millimeter mercury; *OR*, odds ratio.
