# Supplementary material for: Arterial Monitoring in Hypertensive Emergencies: Significance for the Critical Care Resuscitation Unit
Source: West J Emerg Med. 2023 Jul 17;24(4):763–73. doi: 10.5811/westjem.59373 (PMC10393462; doi:10.5811/westjem.59373)
Supplement: Supplementary file 1 [file wjem-24-763-s001.pdf]

Appendix 1. List of independent variables included in multivariate logistic regressions.

Continuous variables
- Age, years
- Body Mass Index

Categorical variables
- Sex (male = 0, female = 1)
- Arterial Catheter location (right = 1, left = 0)
- Comorbidities (yes = 1, no = 0)
  - Diabetes mellitus
  - Hypertension
  - Coronary Artery Disease
  - Peripheral Artery Disease
  - (Any) Kidney Disease
- Diagnosis (yes = 1, no = 0)
  - Acute aortic disease (Aortic Aneurysm, Type A and B Aortic Dissection)
  - Ischemic Stroke (with or without TPA)
  - Spontaneous ICH (Subarachnoid Hemorrhage, Intracerebral Hemorrhage with or without Intraventricular Hemorrhage)
